# Generation of Germline-Competent Rat Induced Pluripotent Stem Cells

**DOI:** 10.1371/journal.pone.0022008

**Published:** 2011-07-15

**Authors:** Sanae Hamanaka, Tomoyuki Yamaguchi, Toshihiro Kobayashi, Megumi Kato-Itoh, Satoshi Yamazaki, Hideyuki Sato, Ayumi Umino, Yukiko Wakiyama, Mami Arai, Makoto Sanbo, Masumi Hirabayashi, Hiromitsu Nakauchi

**Affiliations:** 1 Japan Science Technology Agency, Exploratory Research for Advanced Technology (ERATO), Nakauchi Stem Cell and Organ Regeneration Project, Tokyo, Japan; 2 Division of Stem Cell Therapy, Center for Stem Cell Biology and Regenerative Medicine, Institute of Medical Science, University of Tokyo, Tokyo, Japan; 3 Center for Genetic Analysis of Behavior, National Institute for Physiological Sciences, Okazaki, Japan; 4 School of Life Science, The Graduate University for Advanced Studies, Okazaki, Japan; Instituto de Medicina Molecular, Portugal

## Abstract

**Background:**

Recent progress in rat pluripotent stem cell technology has been remarkable. Particularly salient is the demonstration that embryonic stem cells (ESCs) in the rat (rESCs) can contribute to germline transmission, permitting generation of gene-modified rats as is now done using mouse ESCs (mESCs) or mouse induced pluripotent stem cells (iPSCs; miPSCs). However, determinations of whether rat iPSCs (riPSCs) can contribute to germ cells are not published. Here we report the germline competency of riPSCs.

**Methodology/Principal Findings:**

We generated riPSCs by transducing three mouse reprogramming factors (Oct3/4, Klf4, and Sox2) into rat somatic cells, followed by culture in the presence of exogenous rat leukemia inhibitory factor (rLIF) and small molecules that specifically inhibit GSK3, MEK, and FGF receptor tyrosine kinases. We found that, like rESCs, our riPSCs can contribute to germline transmission. Furthermore we found, by immunostaining of testis from mouse-rat interspecific chimeras with antibody against mouse vasa homolog, that riPSCs can contribute to embryonic development with chimera formation in mice (rat-mouse interspecific chimeras) and to interspecific germlines.

**Conclusions/Significance:**

Our data clearly demonstrate that using only three reprogramming factors (Oct3/4, Klf4, and Sox2) rat somatic cells can be reprogrammed into a ground state. Our generated riPSCs exhibited germline transmission in either rat-rat intraspecific or mouse-rat interspecific chimeras.

## Introduction

Mouse embryonic stem cells (ESCs), first established in 1981, were originally generated from the inner cell mass of mouse blastocysts. Because they are pluripotent, have potentially unlimited capacity for self-renewal, and can contribute to transmitted germlines (exhibit germline competency), mouse ESCs (mESCs) have constituted powerful tools when generating genetically modified mice to understand gene functions and to create mouse models for human diseases [1]. In 2006, Yamanaka et al. reported the generation of pluripotent stem cells from mouse somatic cells by the enforced expression of four transcription factors (Oct3/4, Sox2, c-Myc, and Klf4) selected from genes known to be expressed in ESCs. They referred to these cells as induced pluripotent stem cells (iPSCs). Like mESCs, which they resemble, mouse iPSCs (miPSCs) express alkaline phosphatase (ALP), can generate chimeric mice, and can take part in germline transmission [2] [3]. The discovery of iPSCs, a great step forward in stem-cell research, holds out the promise of development of novel therapeutic strategies by generating iPSCs from patients. Since 2006, besides mESCs and miPSCs, pluripotent stem cells from several mammalian species have been established, including rat [4] [5] [6] [7], rabbit [8] [9], pig [10] [11], monkey [12] [13] [14], and human [15] [16] [17]. Although such iPSCs express a panel of pluripotency markers like SSEA-1, 3, 4, ALP, and Oct3/4, only mESCs and miPSCs can generate chimeras. However, remarkable progress has been made recently, and rat pluripotent stem cell technology now is capable of generating chimeric rats. In 2008, Ying et al. reported establishment of rat ESCs (rESCs) with use of two or three kinds of kinase inhibitors, including glycogen synthase kinase 3 (GSK3) inhibitor, mitogen-activated protein kinase kinase (MEK) inhibitor, and fibroblast growth factor (FGF) receptor tyrosine kinase inhibitor in the culture medium. These inhibitors have been thought to maintain a ground state of pluripotency in mESCs [18] and can support efficient derivation and maintenance of rESCs, permitting generation of chimeric rats, with germline transmission [19] [20]. Shortly after establishment of rESCs, rat iPSCs (riPSCs) also were successfully established. As riPSCs can differentiate into all three germ layers *in vitro* and *in vivo* [21] [22], and can contribute to generating chimeric rat [16]. However, germline competency of riPSCs has not been reported to date.

The rat has been used as a model for studies of physiology, pharmacology, toxicology, nutrition, behavior, immunology, and neoplasia. Its size, its ease of manipulation, and the availability of many kinds of spontaneous models for diseases such as hypertension and diabetes have made the rat the preferred choice for most of these fields, while the mouse has become the leading mammal for experimental genetics. To extend experimental genetics into the rat could be of great value: Generation of iPSCs from disease-model rats could help clarify the pathogenesis of various disorders, particularly if germline-competent iPSCs are necessary to demonstrate curative effects, as when the mutated gene that is responsible for disease can be corrected in riPSCs by homologous recombination and the curative effect can be observed in F1 rats derived from gene-corrected riPSCs.

Here we report for the first time that adding two or three kinds of kinase inhibitor and rLIF to culture medium can generate germline-competent riPSCs. Moreover, we generated reprogrammable rats from riPSCs that were created by infection of three reprogramming factors (*Oct3/4*, *Klf4*, and *Sox2*) via an inducible lentiviral vector.

## Results

### Generation of riPSCs from rat embryonic fibroblasts

**Figure 1:**
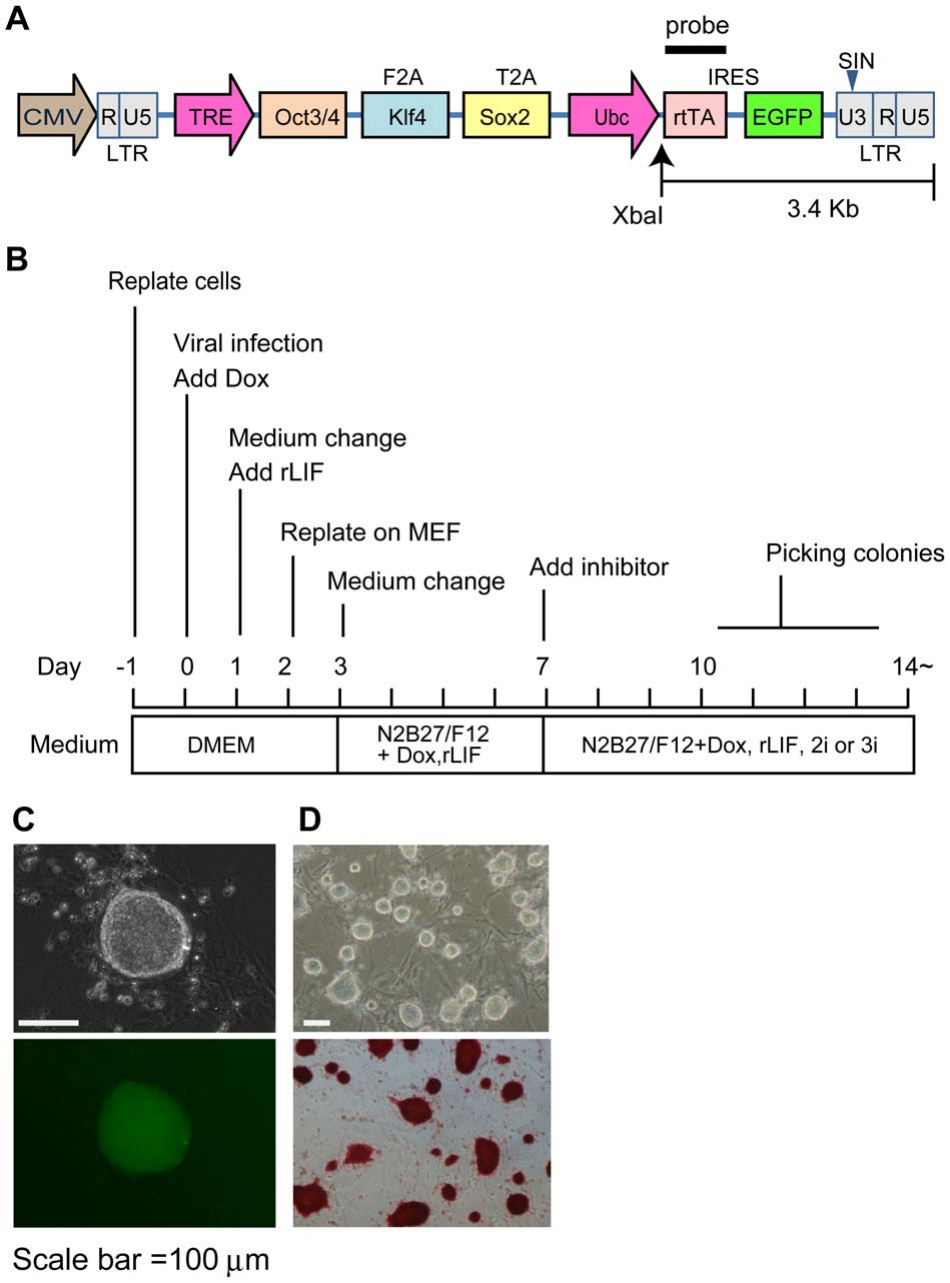
Generation of rat iPS cells from rat embryonic fibroblasts. (A) Schema of inducible lentivirus vector. XbaI enzyme restriction site exists between Ubiquitin-C (*Ubc*) promoter and reverse tet transactivator (rtTA) element. (B) Schematic time schedule of riPSCs generation. (C)–(D) Morphology of generated riPSCs (passage P0); bright field and fluorescent field (C). ALP staining after clonal passage (D).

To generate germline-competent riPSCs, we initially infected Wistar (WI) or Dark Agouti (DA) rat embryonic fibroblasts (REFs) from embryonic day 14.5 or 15.5 (E14.5 or E15.5) with a lentiviral vector carrying three mouse reprogramming factors (*Oct3/4*, *Klf4*, and *Sox2*) controlled by a tetracycline-responsive regulatory element and with *Ubc*-promoter - driven reverse tet transactivator (rtTA) and EGFP (Fig. 1A). We added doxycycline (Dox) to the culture medium on the day of infection (day 0), with rat LIF (rLIF) added to medium on day 1.

Infected REFs were seeded onto mitomycin-C treated mouse embryonic fibroblasts (MEFs) and the medium was changed to serum-free medium (N2B27/F12) containing Dox and rLIF on day 3. On day 7 MEK inhibitor (PD0325901) and GSK3 inhibitor (CHIR99025; 2i) or 2i and FGF receptor inhibitor (SU5409; 3i) were added to the medium (Fig. 1B).

Morphologically ES-like colonies appeared from day 10. They expressed *EGFP* and were of typical dome shape (Fig. 1C). Independent colonies were selected and each colony was expanded in N2B27/F12 medium containing rLIF, 2i, or 3i and with or without Dox. These riPSCs could be sustained for over 25 passages. All ES-like colonies stained for ALP, indicating that generated riPSCs were pluripotent (Fig. 1D). Finally we isolated several riPS clones from both WI and DA fibroblast and chose six clones from WI (T1-3 (referred as riPS#3 in our previous report [23]), T1-4, T1-15, T3-2, T3-3 and T3-11) and two clones from DA (T4-27 and T4-30) for further analysis.

### Pluripotency of iPS cells

**Figure 2:**
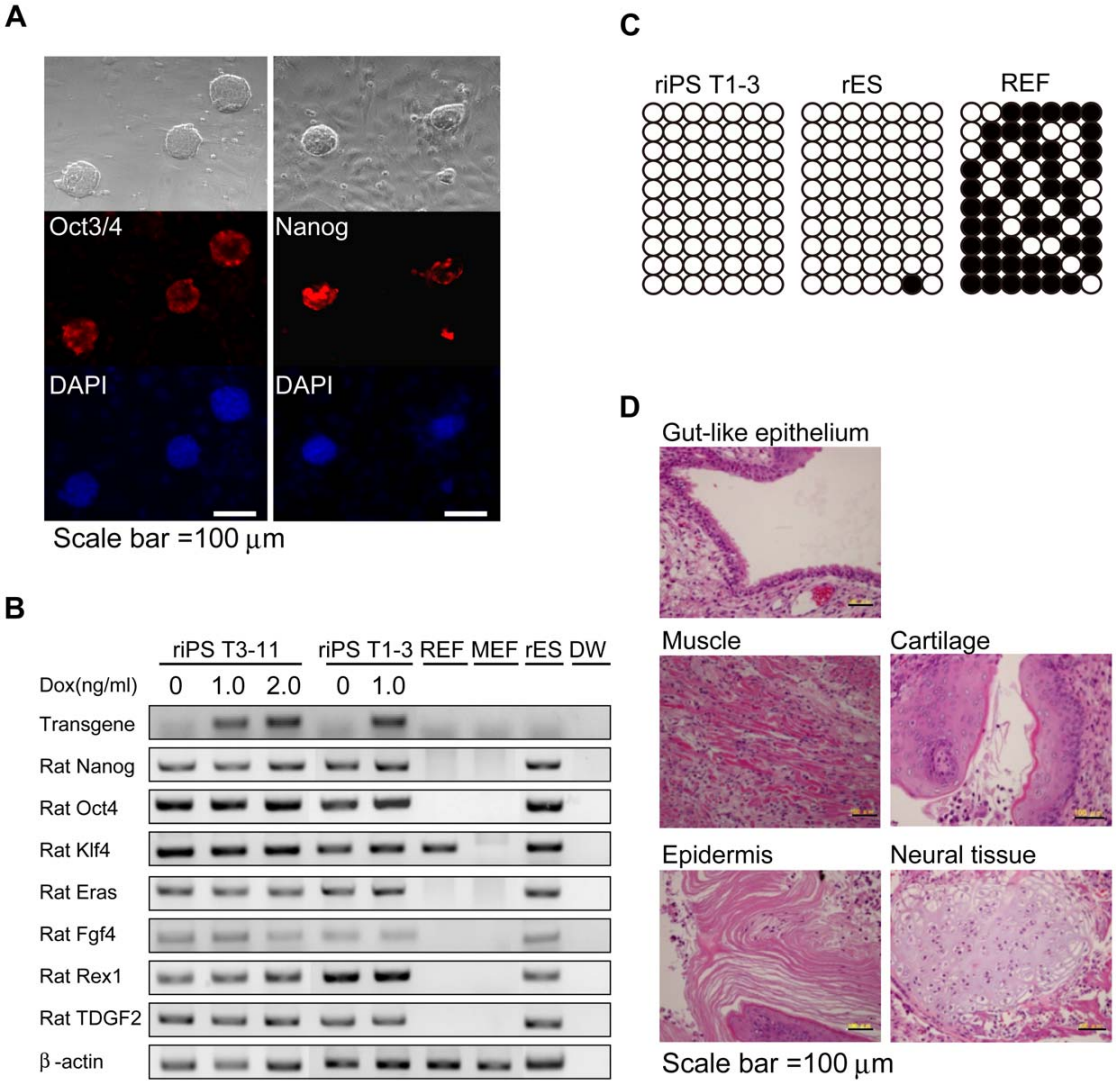
Characterization of rat iPS cells. (A) Immunofluorescence staining for Oct3/4 and Nanog in rat iPS cells (riPSCs). (B) RT-PCR analysis of transgene *T2A-Sox2* and endogenous ES marker genes with or without doxycycline culture. RiPSC clones (riPSCs WI T3-11 and T1-3) express ESC markers. Rat and mouse embryonic fibroblast cells (REFs or MEFs) were used as negative controls. β-actin was used as a loading control. (C) Bisulfite genomic sequencing of the distal enhancer region of *Oct3/4*. Open and closed circles indicate unmethylated and methylated CpGs, respectively. (D) Hematoxylin/eosin staining of teratoma derived from rat iPS cells. Teratoma is composed of various types of tissues: Gut-like epithelium (endoderm), muscle and cartilage (mesoderm), epidermis, and neural tissue (ectoderm).

To evaluate the pluripotency of riPSCs further, we immunostained riPSCs with antibodies against *Nanog* and *Oct3/4* (markers of pluripotency) [16] [24] [25]. RiPSCs expressed both *Nanog* and *Oct3/4* (Fig. 2A). We also examined by RT-PCR the expression of pluripotency marker genes, including *Oct3/4*, *Klf4*, *Fgf4*, *Eras*, *Rex1*, and *Tdgf2*. To detect transgene expression, we designed the primers to amplify sequence between *T2A* and *Sox2*. As shown in Fig. 2B, transgene expression was detected only under with Dox addition, indicating that expression via the lentiviral vector was tightly controlled by a tetracycline-responsive element. We also found that pluripotent marker genes were expressed at levels comparable to those in rESCs; the presence of Dox made no difference (Fig. 2B). The distal enhancer of *Oct3/4* expression reportedly is un-methylated in ESCs (a feature of pluripotency) [26]. To analyze DNA methylation status in riPSCs, we conducted bisulfite-sequencing analysis of genomic regions within the distal enhancer of *Oct3/4* in riPSCs and rESCs. In rESCs and riPSCs the distal enhancer of *Oct3/4* was largely unmethylated, consistent with results from immunostaining and RT-PCR (Fig. 2C). By contrast, in REFs, where *Oct3/4* expression was silenced, the *Oct3/4* enhancer was highly methylated (Fig. 2C).

Next we examined the capacity of riPSCs for *in vivo* differentiation. We injected 0.5–1×10^6^ riPSCs into testis of non-obese diabetic/severe combined immune deficient mice and assessed teratoma formation, with microscopy of hematoxylin/eosin (HE) - stained sections, 4 to 10 weeks after injection. Tumors reached 15 mm or more in diameter and included elements derived from three germ layers (muscle, cartilage, neural tissues, epidermis, gut-like tissues). These results indicate that our riPSCs had been reprogrammed into a pluripotent state and, like ESCs, possessed the capacity for differentiation into three germ layers (Fig. 2D).

### Generation of chimeric rats

**Figure 3:**
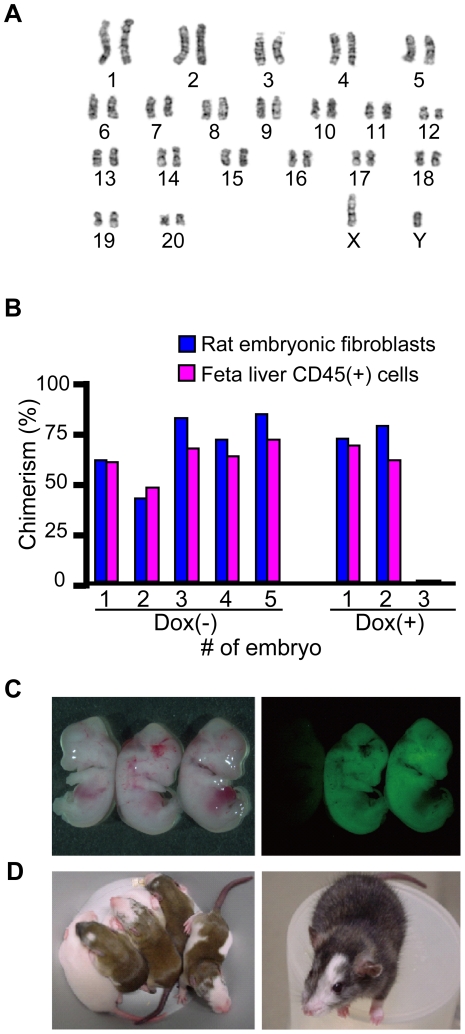
Generation of chimeric rat derived from rat iPS cells. (A) Cytogenetic analysis, riPSCs; G-band staining. Representative data (WI riPSC clone T3-11, passage 7) indicate a chromosomal number of 42, including an XY gender chromosome. (B) Efficiency of chimera rat generation in culture, with or without doxycycline. (C)–(D) Generation of chimeric rat. (C) Analysis of E15.5 material derived from WI riPSCs injected into WI blastocysts. EGFP indicates riPSC-derived cells. (D) Chimeric rats derived from DA riPSCs injected into WI blastocysts. Brown coat color indicates derivation from DA riPSCs. One-week-old pups; left pup (white) not chimeric (left panel). Three-month-old chimeric rat; eye color also is brown (right panel).

Before attempting to generate chimeric rats, we evaluated riPSC karyotypes; they were normal (42XY; Fig. 3A). However, trisomy of chromosome 9 was encountered in clones T1-3, T1-4 and T1-15 (rate: 2/50, 6/50 and 3/50 respectively) cultured with 3i (Figure S1), as observed [20]. As no chromosome abnormality was found in clones T3-2, T3-3 and T3-11 derived from cells cultured with 2i, we continued to culture riPSCs with 2i thereafter. Next we examined the effect of Dox in culture medium on generation of chimeras. WI riPSCs cultured with Dox or without Dox were injected into (WI×WI) rat blastocysts. EGFP expression was assessed by fluorescence microscopy of E15.5 embryos. Using riPSCs cultured with Dox until blastocyst injection, chimeric rat embryos were generated at a ratio of 66%; without Dox, the rate was 100%. Because half life of Dox is 24 hrs, transgene expression continues in embryo after blastocyst injection. This continuous expression of transgene in embryo might influence the chimera contribution. Therefore, for efficient generation of chimeric rat, we cultured riPSCs without Dox thereafter (Fig. 3B, Table 1).

**Table 1:** Chimera rat generation from rat iPS cells.

Cell line	Wistar T1-3 42XY	Wistar T1-3 42XY	Wistar T1-3 42XY	Wistar T1-3 42XY	Wistar T3-11 42XY	DA T4-27 42XY	DA T4-30 42XY
Passage number	P20	P14	P14	P14	P15	P7	P8
Dox added	(−)	(−)	(+)	(−)	(−)	(−)	(−)
Host blastcyst	WI×DA	WI×WI	WI×WI	WI×WI	WI×WI	WI×WI	WI×WI
Number of Blastcysts injected	43	13	12	39	33	28	42
Developed to fetus	N/A	5 (38%)	3 (25%)	N/A	N/A	N/A	N/A
Developed to pups	4 (9%)	N/A	N/A	18 (46%)	13 (40%)	6 (21%)	26 (62%)
Number of chimeras	2 (50%) 1M,1F	5 (100%)	2 (66%)	11 (61%) 4M, 7F	7 (54%)4M, 3F	2 (33%) 1M, 1F	15*1 (58%) 7M, 7F
Number of germline chimeras	N/A	N/A	N/A	2/3 M	1/4 M	0/1 M	2/5 M

N/A: not applicable.

*1: One chimera died before weaning.

Next we injected riPSCs from clone T1-3 into (DA×WI) F1 blastocysts to permit identification of chimeras by coat color. Using foster mothers and 43 blastocysts into which riPSCs were injected, we obtained four pups of which two were chimeras (Table 1).

Because rat (DA×WI) F1 blastocysts might have potentially low reproduction ability, we then tried to generate chimeras by using rat (WI×WI) blastocysts, detecting chimerism by evaluating expression of EGFP. We obtained 11 chimeras in 18 liveborn pups using clone T1-3 and 7 chimeras in 13 liveborn pups using clone T3-11. We also injected T4-27 and T4-30 into rat (WI×WI) blastcysts and obtained 2 chimeras in 6 liveborn pups and 15 chimeras in 26 liveborn pups respectively (Fig. 3C, Table 1).

These results indicate that riPSCs generated in medium containing 2i can contribute to generating chimeric rats.

### Germline transmission of riPSCs

To address whether these riPSCs could contribute to germline transmission, generated male chimeric rats were mated with wild-type female WI rats.

**Figure 4:**
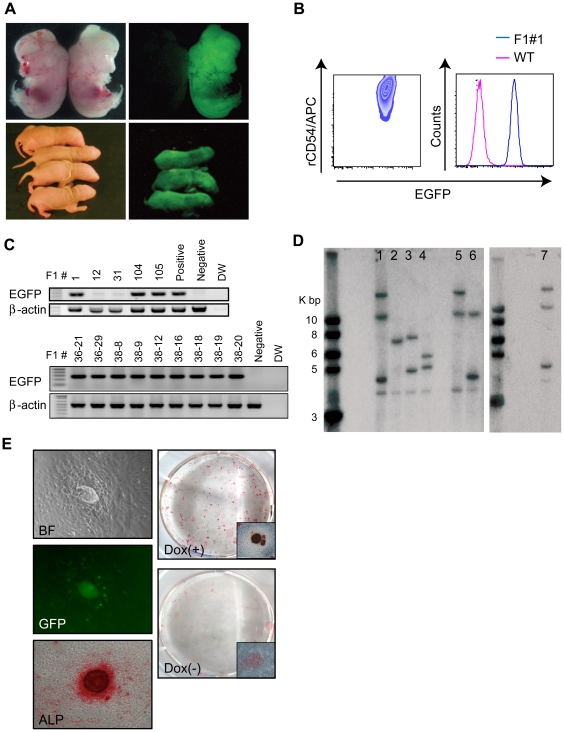
Germline transmission of riPSCs. (A) EGFP of offspring (E15.5; neonate) of chimeric rats. (B) FACS analysis of rat embryonic fibroblasts (REFs) established from F1 rats. (C) Genomic PCR, F1 rats. Some F1 rats harbored *EGFP*. Genomic DNA was obtained from F1 REFs or from tail. (D) Provirus copy numbers determined by Southern blot analysis. Lane1: WI riPSCs, clone T1-3; 2: WI riPSCs, clone T3-11; 3: DA riPSCs, clone T4-27; 4: DA riPSCs, clone T4-30; 5–7: EGFP-expressing F1 rats. (E) REFs established from F1 rats using Dox were reprogrammable: Re-generated riPSCs expressed EGFP and ALP.

We obtained three embryos (E15.5) and eight pups that expressed EGFP from three chimeric rats derived from riPSC clones T1-3 or T3-11 (Fig. 4A, Table 1, Table S1). FACS analysis revealed that REFs established from F1 rats also expressed EGFP (Fig. 4B) and, as shown in Fig. 4C, proviral DNA was detected by genomic PCR. For further confirmation of the germline transmission of riPSCs, we attempted to regenerate riPSCs from fibroblasts of F1 rats. Because the clone T1-3 riPSCs from which F1 rats were generated were established using a Dox-inducible lentiviral vector, exposure to Dox should allow riPSCs to regenerate from F1 rat somatic cells. REFs from F1 rats seeded on MEFs and cultured in medium containing Dox. ESC-like colonies began to appear from day 10 and by day 16 and 2,000 REFs had given rise to 16.6 ES-like colonies (triplicate assay; Fig.4E). The frequency of regeneration, calculated as the number of colonies/2000×100 was 1% on day 16 and 4.7% on day 20.

Chimeras derived from riPSC clones T4-27 and DA T4-30 had no offspring that expressed EGFP or had black eyes (Because eye color of DA is black and of WI is red, we can identify F1 rat by black eye color).

However, we found that the seminiferous tubules of these DA chimeras expressed EGFP (Figure S2A). Moreover, immunostaining revealed that germ cells in testis from chimeric rats which were derived from riPSC clone T4-30, contained mouse vasa homolog (MVH) and EGFP double positive cells, indicating that clone T4-30 riPSCs can contribute to germ line transmission (Figure S2B). On the other hand, we could not detect MVH and EGFP double positive cells in testis from chimeras derived from clone T4-27 riPSCs (data not shown).

To confirm provirus copy number, genomic DNA from riPSCs and from F1 REFs were analyzed by Southern blot (Fig. 4D). Four riPSC lines (lanes 1–4) showed different integration patterns containing one to three transgenes. Progeny derived from clone T1-3 riPSCs (lanes 5–7) carried provirus copies inherited from their riPSCs of origin. Either two or three provirus copies were integrated into the genomes of F1 rats (Table S1).

Tumorigenesis has been observed in chimeric mice (and their offspring) derived from iPSCs generated by transduction of four reprogramming factors (*c-Myc*, *Oct4*, *Sox2*, and *Klf4*), and has been ascribed to reactivation of c-Myc [3]. In our study, however, no tumors developed in chimeric rats or their progeny during observation for over six months.

As a whole, these data show that riPSCs generated by our method can contribute to germline transmission and pose a risk of tumorigenesis lower than that observed using miPSCs generated by a different method.

### Generation of interspecific chimeras between mouse and rat with contribution to germline chimerism

**Figure 5:**
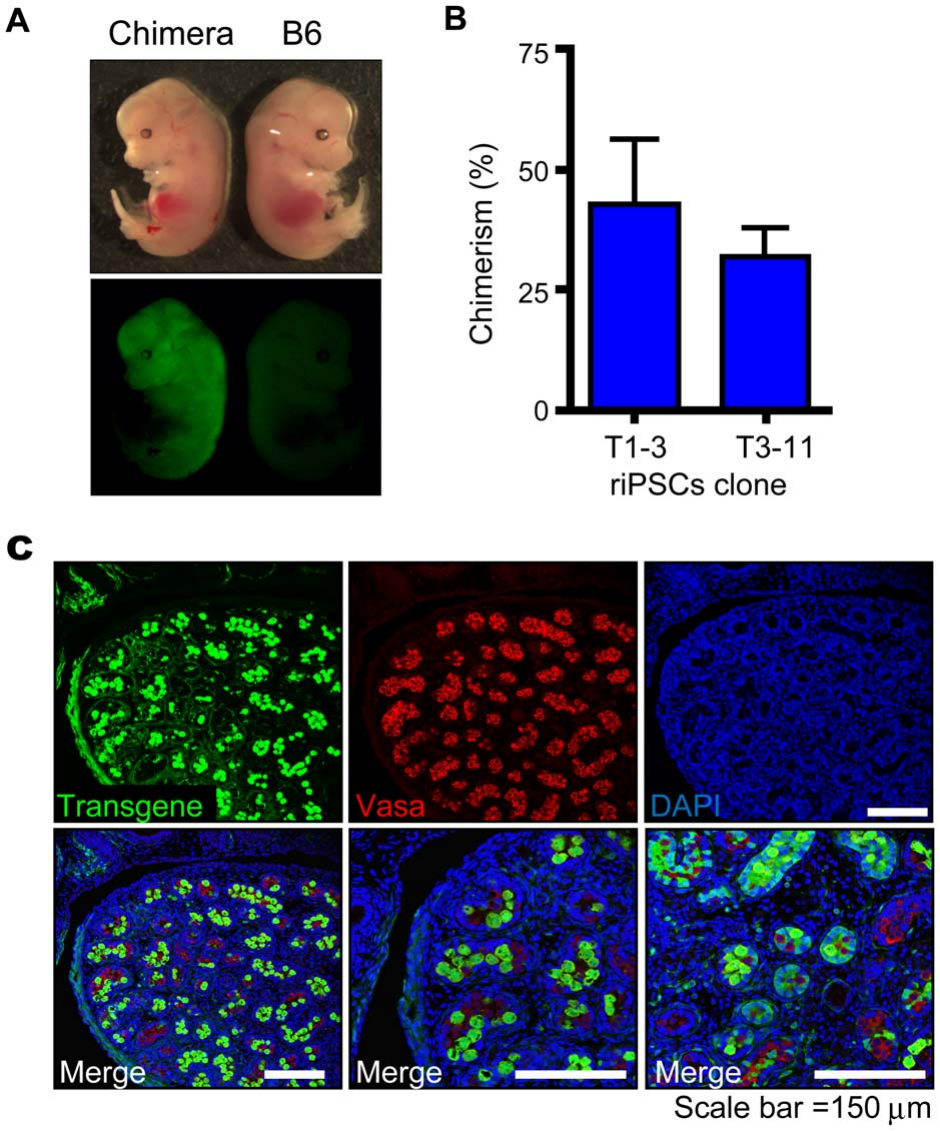
Generation of interspecific chimeras between mouse and rat. (A) RiPSCs injected into mouse blastocysts; analysis, E13.5. EGFP of chimeric mouse (E13.5) generated using rat iPSCs. (B) EGFP (%) of chimerism was shown by FACS analysis of chimeric mouse embryonic fibroblasts (MEFs/REFs) established from F1 rats. (C) Immunostaining of chimeric rats. HMV/DTT (Alexa546) or EGFP fluorescence was observed in testis. EGFP was derived from riPSCs.

We have recently reported successful generation of rat-mouse interspecific chimeras by injection of riPSCs into mouse blastocyst [23]. To assess if the riPSC clones T1-3 or T3-11 can contributed to embryonic development with chimeric formation in wild type mice, we generated rat-mouse interspecific chimeras and analyzed the expression of EGFP in embryonic fibroblasts (Fig. 5A). Embryos derived from both riPSC clones expressed EGFP, indicating that most riPSC clones can form chimeras (Fig. 5B). Moreover, immunostaining found MVH and EGFP double positive cells in testis from interspecific chimeras (n = 2) derived from riPSCs T1-3 (Fig. 5C). These data provide conclusive evidence that these riPSCs are pluripotent and that they can contribute to both interspecific chimera formation and germline transmission.

## Discussion

We here show that infection with a lentiviral vector carrying three mouse reprogramming factors (*Oct4*, *Klf4*, and *Sox2*) and culture in the presence of two kinds of kinase inhibitors (MEK inhibitor and GSK3 inhibitor) permit efficient establishment and maintenance of riPSCs from REFs. Established riPSCs possess all the key features of rESCs, such as expression of pluripotency markers Oct4, Nanog, and E-ras, long-term self-renewal, the capacity to differentiate into derivatives of all three germ layers, and, most importantly, the ability to produce chimerism with high efficiency and to contribute to transmission through the germline.

MiPSCs generated by introducing four reprogramming factors (*Oct3/4*, *Sox2*, *Klf4*, and *c-Myc*) are able to produce germline-competent chimeras.

However, a drawback in this four-factor system is reactivation of the c-Myc retrovirus, which increases tumorigenicity in chimeric mice and their progeny. On the other hand, no tumors were observed in mice derived from iPSCs generated by introducing three factors (*Oct3/4*, *Sox2*, and *Klf4*) [27]. Therefore, we used three factors (*Oct3/4*, *Klf4*, and *Sox2*), eliminating c-Myc, with a special emphasis on escape from tumor development. As expected, we observed no tumorigenesis in chimeric rats or their progeny, with monitoring for over six months.

Two main factors can be conceived to affect the successful generation of germline chimeras. One is the conditions under which riPSCs are generated and cultured. In recent studies by Ping Li et al., rESCs were generated using serum-free N2B27 medium containing mLIF and a combination of two or three kinase inhibitors (2i/3i) [19] [20]. RiPSCs also have been generated using knockout DMEM medium containing knockout serum replacement (KSR), 2i, A83-01 and mLIF [16]. Because successful germline transmission was reported only in the rESCs study, we generated riPSCs using N2B27 medium and the 2i/3i culture method. The other is the interaction between iPSC strain and host blastocyst strain. In this study, high efficiency of germline transmission was achieved by injection of WI riPSCs into WI blastocysts (donor cell : host blastocyst combination WI : WI) and of DA riPSCs into WI blastocysts (DA : WI). Other studies have generated rESC germline chimeras in the combinations DA : F344 [16,19], WI :WI and DA/WI [28], and WI-LEA : WI, WI : WI, WI : LEA, and LEA : WI [29]. So far, only a few combinations of iPSCs strains and blastocyst strains have successfully generated germline chimeras. Although we could not obtain offspring from DA riPSCs, we may need to test different strains of blastocysts for more efficient germline transmission.

As in our previous report of interspecific chimeras [23], the riPSCs generated in this study were able to contribute interspecifically to chimera generation and interspecifically to germ cell lineages, indicating that these riPSCs were highly reprogrammed.

Taken together, our results demonstrate that rat somatic cells can be reprogrammed to ground state with germ line competence by transduction of only three reprogramming factors (*Oct3/4*, *Klf4*, and *Sox2*). We believe that these riPSCs will open a new area of studies using the rat as a useful animal model.

## Materials and Methods

### Cell culture

Rat embryonic fibroblasts (REFs) were derived from embryonic day (E)14.5 or E15.5 Wistar (WI) and Dark agouti (DA) rat embryos. WI and DA rats were purchased from Japan SLC (Shizuoka, Japan). REFs were cultured in Dulbecco's modified Eagle's medium (DMEM; Sigma, St. Louis, MO) supplemented with 10% fetal bovine serum (FBS; Hana-Nesco Bio, Moregate BioTech, Australia), 1% L-glutamine penicillin streptomycin (Sigma, St. Louis, MO). Rat iPS cells (riPSCs) were maintained on mitomycin-c treated mouse embryonic fibroblasts (MEFs) in N2B27 medium (Invitrogen, Carlsbad, CA) [30] supplemented with 1,000 U/ml of rat leukemia inhibitory factor (LIF, ESGRO Millipore, Bedford, MA), 3 µM of GSK3 inhibitor CHIR99021 (Axon Medchem BV, Groeningen, The Netherlands), and 1 µM of MEK inhibitor PD0325901 (Stemgent, Cambridge, MA), with or without FGF receptor inhibitor SU5402 (2 µM; Calbiochem, La Jolla, CA). RiPSCs were trypsinized (0.05% trypsin-EDTA: Sigma, St. Louis, MO) into single cells and plated into new wells with a MEFs feeder every 3 or 4 days. Rat embryonic stem cells (rESCs) were maintained as described protocol [28].

### Lentiviral vector; generation of riPSCs

The cDNAs of mouse *Oct3/4*, *Sox2*, and *Klf4* were inserted into a doxycycline-inducible system lentiviral vector that also included *EGFP* inserted downstream from a Ubiquitin-C promoter.

To establish riPSCs, Wistar and DA REFs were transduced with this lentiviral vector. Two days later, transduced cells were trypsinized and split among MEFs feeder wells. Eight days later (day 10 after transduction), generated colonies were picked up and mechanically dissociated. Dissociated cells (riPSCs) were plated into new wells with a MEFs feeder (96-well plates). The riPSCs uniformly expressed EGFP under the control of the Ubiquitin-C promoter.

### Blastocyst injection and generation of chimeric rats

Chimeric rats were generated by a conventional method: RiPSCs were microinjected into day 4.5 blastocysts of WI×DA-F1 or WI female rats, followed by transfer into host uteri as described. The resultant chimeric rats were mated with WI rats to obtain their progeny [23] [28].

For generation of interspecific chimeras between mouse and rat, we employed conventional method of chimeric mice generation [31]. RiPSCs were microinjected into day 3.5 blastocysts of C57BL/6 or ICR female mice (purchased from SLC Japan), followed by transfer into host uteri as described [23].

### ALP staining and immunostaining

Histochemical ALP assays were conducted with Vector Red Alkaline Phosphatase Substrate Kit I (Vector Laboratories, Burlingame, CA) according to the manufacturer's instructions.

For immunofluorescence assays, cells were fixed in 4% paraformaldehyde for 10 min and washed twice with PBS. The fixed cells were incubated in MAXblocking medium (Active Motif, Carlsbad, CA) for 30 min at room temperature (RT) for blocking. The cells were then incubated with primary antibody for one hour at RT in blocking buffer. Cells were then washed with PBS and incubated with secondary antibody in PBS for 30 min at RT. Thereafter the cells were washed with PBS and 4′,6 -diamidino-2-phenylindole (DAPI) was added for nuclear staining.

Primary antibodies used were rabbit anti-mouse Oct3/4 antibody (Bioworld Technology, Minneapolis, MN, 1∶100) and rabbit anti-mouse Nanog antibody (ReproCELL, Kanagawa, Japan, 1∶100). Secondary antibodies were Alexa Fluor 546 conjugated goat anti-rabbit IgG antibody (1∶300) or Alexa Fluor 546 conjugated goat anti-mouse IgG antibody (1∶300) (Invitrogen, Carlsbad, CA, 1∶300).

Testes were fixed in 4% paraformaldehyde and PBS+sucrose solutions ranging from 10% to 30% (w/v) sucrose before freezing in optimal cutting temperature medium (Tissue Tek, Sakura Finetek, Torrance, CA) and cryostat sectioning. Fixed sections were incubated in MAXblocking medium for 30 min at RT for blocking. Each section was incubated with primary antibody for 1 hr at RT and with secondary antibody for 1 hr at RT. Primary antibodies used were goat anti-GFP (Abcam, Cambridge, MA, 1∶100) and rabbit anti-DDX4/MVH (as a primitive germ cell marker; Abcam, 1∶100). Secondary antibodies were Alexa Fluor 546 conjugated donkey anti-rabbit IgG antibody and Alexa Fluor 488 conjugated donkey anti-goat IgG antibody (Invitrogen, 1∶300). After antibody treatment, sections were mounted with Vectashield (Vector Laboratories), a mounting medium containing DAPI for nuclear counterstaining, and sections were observed under fluorescence microscopy.

### Genomic PCR and RT-PCR

Genomic DNA was extracted using QIAamp DNA Mini Kits (Qiagen, Germantown, MD) or the phenol-chloroform method. PCR was performed in Takara PCR Thermal Cycler Dice® (Takara Bio, Shiga, Japan). Total RNA prepared using the RNAeasy kit (Qiagen) was used as a template for reverse transcription-polymerase chain reaction III (RT-PCR). For genomic PCR and RT-PCR of GFP and β-actin, EX Taq HS (Takara) was used under the following conditions: 94°C for 1 min, followed by 30 or 35 cycles of 94°C for 30 sec, annealing temperature (from 50°C to 62°C) for 30 sec (for genomic PCR) or 1 min (for RT PCR), and 72°C for 30 sec, with a final extension at 72°C for 7 min. PCR products were visualized with ethidium bromide on a 1.2% agarose gel. The primer sequences are listed in Table S2 with annealing temperature and PCR product size.

### Southern blotting

High-molecular-weight DNA was obtained from riPSCs or generated REFs. Genomic DNA was digested with XbaI, electrophoresed (1% agarose gel), transferred to a nylon membrane (Hybond-XL: Amersham Biosciences GE Healthcare, UK), and hybridized to [α-^32^P]dCTP (deoxycytidine 50-triphosphate)-labeled rtTA sequence which is encoded in doxycyclin inducible lentivirus vector.

As the restriction enzyme XbaI cut the vector at one site, the number of fragments hybridized with the probe is considered to be the number of provirus copies integrated into the host genome. Hybridization signals were detected with an auto image analyzer (FLA 5100, Fuji Film, Tokyo, Japan) after exposure to an imaging plate.

### Bisulfite sequencing

Genomic DNA was extracted with a genomic DNA purification kit (QIAGEN).

200 ng of genomic DNA from each sample was treated with a Methylamp DNA modification sample kit (Epigentek, Brooklyn, NY) to convert the unmethylated cytosine to uracil according to the manufacturer's instructions. The promoter region of *Oct4* was amplified by PCR using EX Taq HS (Takara), cloned into the pCR 2.1 vector (Invitrogen), and sequenced with M13 reverse and forward primers. PCR primers are listed in Table S2.

### Karyotype analysis

Karyotype analysis was performed with standard methods (Nihon Gene Research Laboratories, Miyagi, Japan).

### Teratoma formation

A suspension of 0.5∼1.0×10^6^ single cells was injected intra-testis into each testis of 6- to 10-week-old non-obese diabetic/severe combined immune deficient mice. After 4–8 weeks, tumors were harvested and processed for hematoxylin/eosin staining.

### Cell surface analysis

To detect chimerism, REFs and fetal liver cells from riPSC-derived chimeric rats and offspring (E14.5–15.5) were stained with phycoerythrin (PE) - conjugated mouse anti-rat CD45 antibody (OX-1, Biolegend), biotin-conjugated mouse anti-rat CD54 antibody (ICAM-1, 1A29, BD Biosciences PharMingen, San Diego, CA) and Alexa Fluor 647-conjugated goat anti-mouse IgG antibody (BD Biosciences PharMingen). Peripheral blood cells were also stained with PE-conjugated mouse anti-rat CD45 antibody. All stained cells were analyzed by cytometry using FACSCanto II (BD Bioscience).

All experiments were performed under institutional guidelines.

Animal experiments were performed with approval of the Institutional Animal Care and Use Committee of the Institute of Medical Science, University of Tokyo (permit numbers: A09-29, A09-30, A10-23) and the Institutional Animal Care and Use Committee of the National Institute for Physiological Sciences (permit number: 11A022).

## Supporting Information

Figure S1 **Karyotype analysis of cells cultured with 3i.** Cytogenetic analysis, riPSCs; G-band staining. Representative data of WI riPSCs, clone T1-3, at passage 20 when cultured with 3i-medium indicate trisomy of chromosome 9 in 2 out of 50 cells, including an XY gender chromosome, but within normal range of polyploidy. A few cells exhibiting trisomy of chromosome 9 in another two clones were cultured with 3i-medium.
(TIF)

Figure S2 **Phenotype of chimeric rat derived from DA riPSCs, clone T4-30.** (A) EGFP expression in testis. (B) Immunostaining of chimeric rat testis. HMV/DTT (Alexa546) or EGFP fluorescence was observed in testis. EGFP was derived from riPSCs.
(TIF)

Table S1 **Summary of offspring-rat generation from riPSCs.**
(DOC)

Table S2 **Summary of primer sequences.**
(DOCX)
