# Supplementary figures and images for: Generation of Germline-Competent Rat Induced Pluripotent Stem Cells
Source: PLoS One. 2011 Jul 15;6(7):e22008. doi: 10.1371/journal.pone.0022008 (PMC3137610; doi:10.1371/journal.pone.0022008)

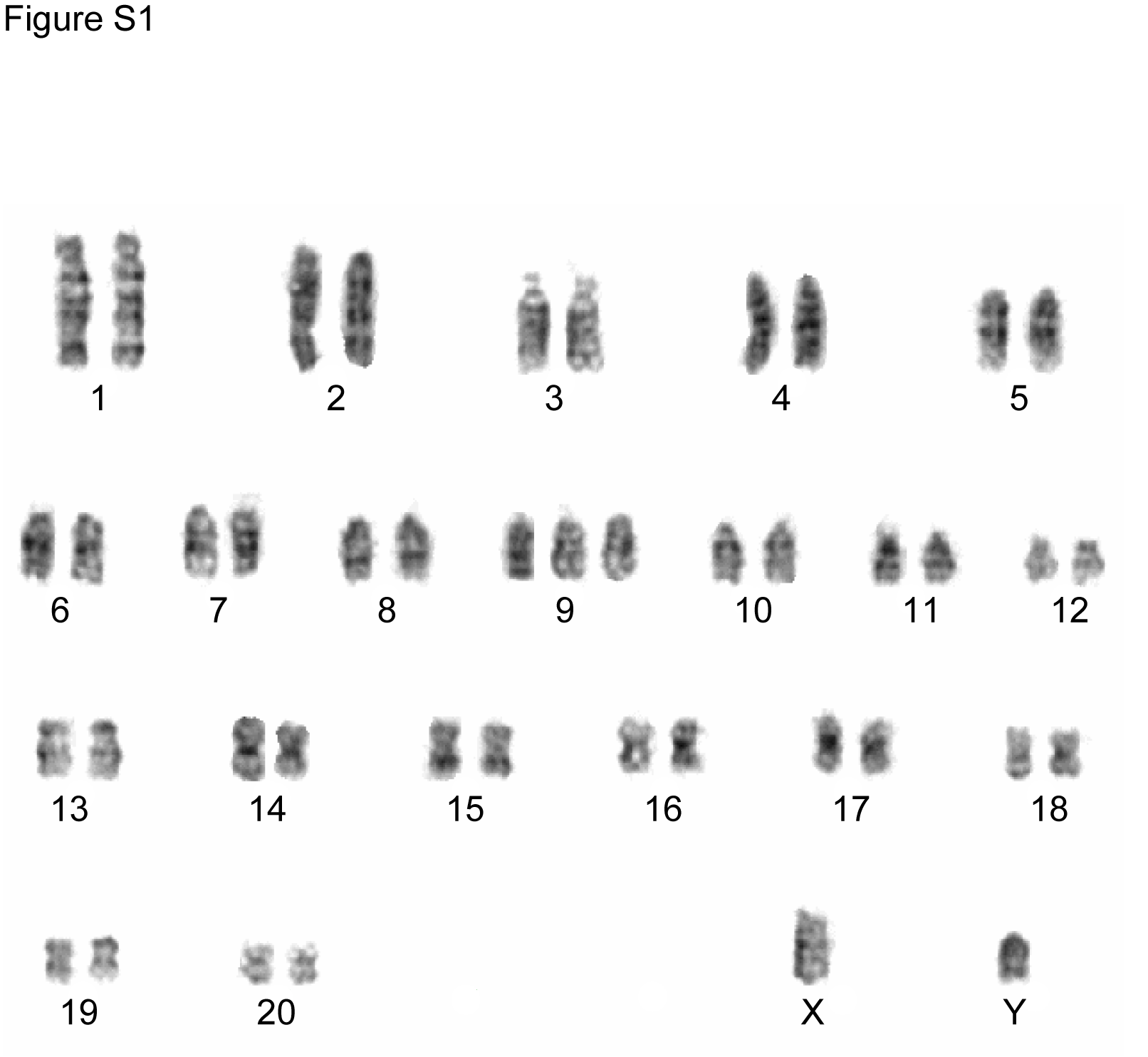

Supplement: Figure S1 — Karyotype analysis of cells cultured with 3i. Cytogenetic analysis, riPSCs; G-band staining. Representative data of WI riPSCs, clone T1-3, at passage 20 when cultured with 3i-medium indicate trisomy of chromosome 9 in 2 out of 50 cells, including an XY gender chromosome, but within normal range of polyploidy. A few cells exhibiting trisomy of chromosome 9 in another two clones were cultured with 3i-medium. (TIF) [file pone.0022008.s001.tif]

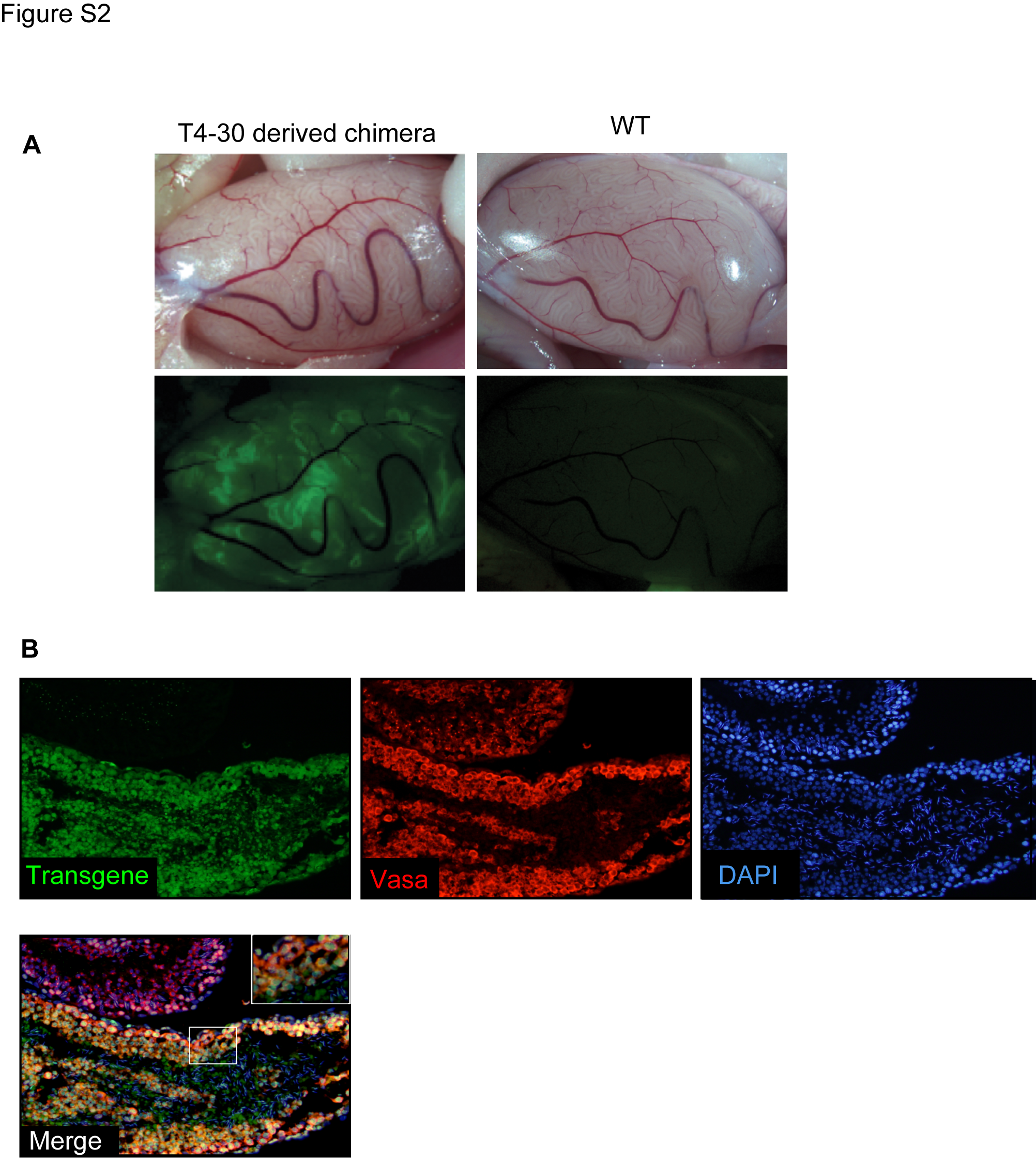

Supplement: Figure S2 — Phenotype of chimeric rat derived from DA riPSCs, clone T4-30. (A) EGFP expression in testis. (B) Immunostaining of chimeric rat testis. HMV/DTT (Alexa546) or EGFP fluorescence was observed in testis. EGFP was derived from riPSCs. (TIF) [file pone.0022008.s002.tif]
