# Supplementary material for: Generation of Germline-Competent Rat Induced Pluripotent Stem Cells
Source: PLoS One. 2011 Jul 15;6(7):e22008. doi: 10.1371/journal.pone.0022008 (PMC3137610; doi:10.1371/journal.pone.0022008)
Supplement: Table S1 — Summary of offspring-rat generation from riPSCs. (DOC) [file pone.0022008.s003.doc]

| riPS cell lines and Germline contributed Offspring pups | Macroscopic Fluorescent | Facs Analysis | PCR GFP | Copy Number |
| --- | --- | --- | --- | --- |
| riPS T1-3 | (+) | (+) | (+) | 3 |
| riPS T3-11 | (+) | (+) | (+) | 1 |
| T1-3 F1#1 (Embryo) | (+) | (+) | (+) | 3 |
| T1-3 F1#104 (Embryo) | (+) | (+) | (+) | 2 |
| T1-3 F1#105 (Embryo) | (+) | (+) | (+) | 2 |
| T1-3 F1 #36-21 (Neonate) | (+) | N/A | (+) | N/A |
| T1-3 F1 #36-29 (Neonate) | (+) | N/A | (+) | N/A |
| T1-3 F1 #38-8 (Neonate) | (+) | N/A | (+) | N/A |
| T1-3 F1 #38-9 (Neonate) | (+) | N/A | (+) | N/A |
| T1-3 F1 #38-12 (Neonate) | (+) | N/A | (+) | N/A |
| T1-3 F1 #38-16(Neonate) | (+) | N/A | (+) | N/A |
| T1-3 F1 #38-18 (Neonate) | (-) | N/A | (+) | N/A |
| T1-3 F1 #38-19 (Neonate) | (-) | N/A | (+) | N/A |
| T1-3 F1 #38-20 (Neonate) | (+) | N/A | (+) | N/A |
| T3-11 F1 #40-14 (Neonate) | (+) | N/A | N/A | N/A |

**Table S1 Offspring-rat generation from rat iPS cells**

N/A: not applicable.
