# Supplementary material for: Generation of Germline-Competent Rat Induced Pluripotent Stem Cells
Source: PLoS One. 2011 Jul 15;6(7):e22008. doi: 10.1371/journal.pone.0022008 (PMC3137610; doi:10.1371/journal.pone.0022008)
Supplement: Table S2 — Summary of primer sequences. (DOCX) [file pone.0022008.s004.docx]

|  | **Primer sequences** | **Product size(bp)** | **Annealing temperature** |  |
| --- | --- | --- | --- | --- |
| Transgene T2A Fw | GGAAGTCTGCTAACATGCGGTG | 200 | 54℃ |  |
| Transgene mSox2 Rv | GGCCATACCATGAAGGCGTTCAT |  |  |  |
| Rat Oct4 RT FW | CGAGGCCTTTCCCTCTGTTCCT | 119 | 62℃ |  |
| Rat Oct4 RT RV | TCTCTTTGTCTACCTCCCTTCCTTGC |  |  |  |
| Rat Klf4 RT Fw | CAGACCTGGAAAGTGGTGG | 283 | 58℃ |  |
| Rat Klf4 RT Rv | ACCTGTGTTGCCCGCAGCC |  |  |  |
| Rat Sox2 RT FW | GGCCATTAACGGCACACTGCC | 120 | 62℃ |  |
| Rat Sox2RT RV | TTACTCTCCTCTTTTGCACCCCTCC |  |  |  |
| Rat Rex-1 RT Fw | AAATCATGACGAGGCAAGGC | 350 | 56℃ |  |
| Rat Rex-1 RT Rv | TGAGTTCGCTCCAACAGTCT |  |  |  |
| Rat TDGF2 RT Fw | AACACCAACAATATTTTATGTGGCC | 511 | 56℃ |  |
| Rat TDGF2 RT Rv | TCATTTCTAGGAAAAGGCAGATGC |  |  |  |
| Rat Eras RT Fw | CGAGCGGTGTGGGTAAAAGTG | 501 | 50℃ |  |
| Rat Eras RT Rv | GGTGTCGGGTCTTCTTGCTTG |  |  |  |
| Rat Fgf4 RT Fw | CGGGGTGTGGTGAGCATCTTC | 202 | 50℃ |  |
| Rat Fgf4 RT Rv | CCTTCTTGGTCCGCCCGTTC |  |  |  |
| EGFP Fw | ATGGTGAGCAAGGGCGAG | 249 | 58℃ |  |
| EGFP Rv | AGTCGTGCTGCTTCATGTGG |  |  |  |
| β-actin FW | CATGGCATTGTGATGGACT | 427 | 53℃ |  |
| β-actin Rv | ACGGATGTCAACGTCACACT |  |  |  |
| Bisulfate sequence |  |  |  |  |
| Rat Oct4 bisulfite FW | ATGGGATTTTGGAGGATTTTTAG | 206 | 53℃ |  |
| Rat Oct4 bisulfite RV | CTCAAACCCAAATACCCCTACTT |  |  | |

**Table S2 Primer sequences**
